# Supplementary material for: A male-derived volatile sex pheromone in Caenorhabditis nematodes identified through its mimicry by a predator
Source: Commun Biol. 2026 Jul 1;9:1382. doi: 10.1038/s42003-026-10575-4 (PMC13687015; doi:10.1038/s42003-026-10575-4)
Supplement: Supplementary file 1 [file 42003_2026_10575_MOESM1_ESM.pdf]

Supplementary Information

A male-derived volatile sex pheromone in *Caenorhabditis* nematodes identified through its mimicry by a predator

Authors: Matthew R. Gronquist[1], Xuan Wan[2], Daniel Leighton[3], Yuki Togawa[4], Marika Sagawa[4], Paul W. Sternberg[2]*, Frank C. Schroeder[5], Ryoji Shinya[4]*

Affiliations:

1. Department of Chemistry and Biochemistry, State University of New York at Fredonia, USA
2. Division of Biology and Biological Engineering, California Institute of Technology, USA
3. Current address: Department of Human Genetics, Department of Biological Chemistry, and Howard Hughes Medical Institute, University of California, Los Angeles, USA
4. School of Agriculture, Meiji University, Japan
5. Boyce Thompson Institute and Department of Chemistry and Chemical Biology, Cornell University, USA

*Corresponding authors

Emails

Paul Sternberg; pws@caltech.edu; 0000-0002-7699-0173

Ryoji Shinya; shinya@meiji.ac.jp; 0000-0002-2450-3045

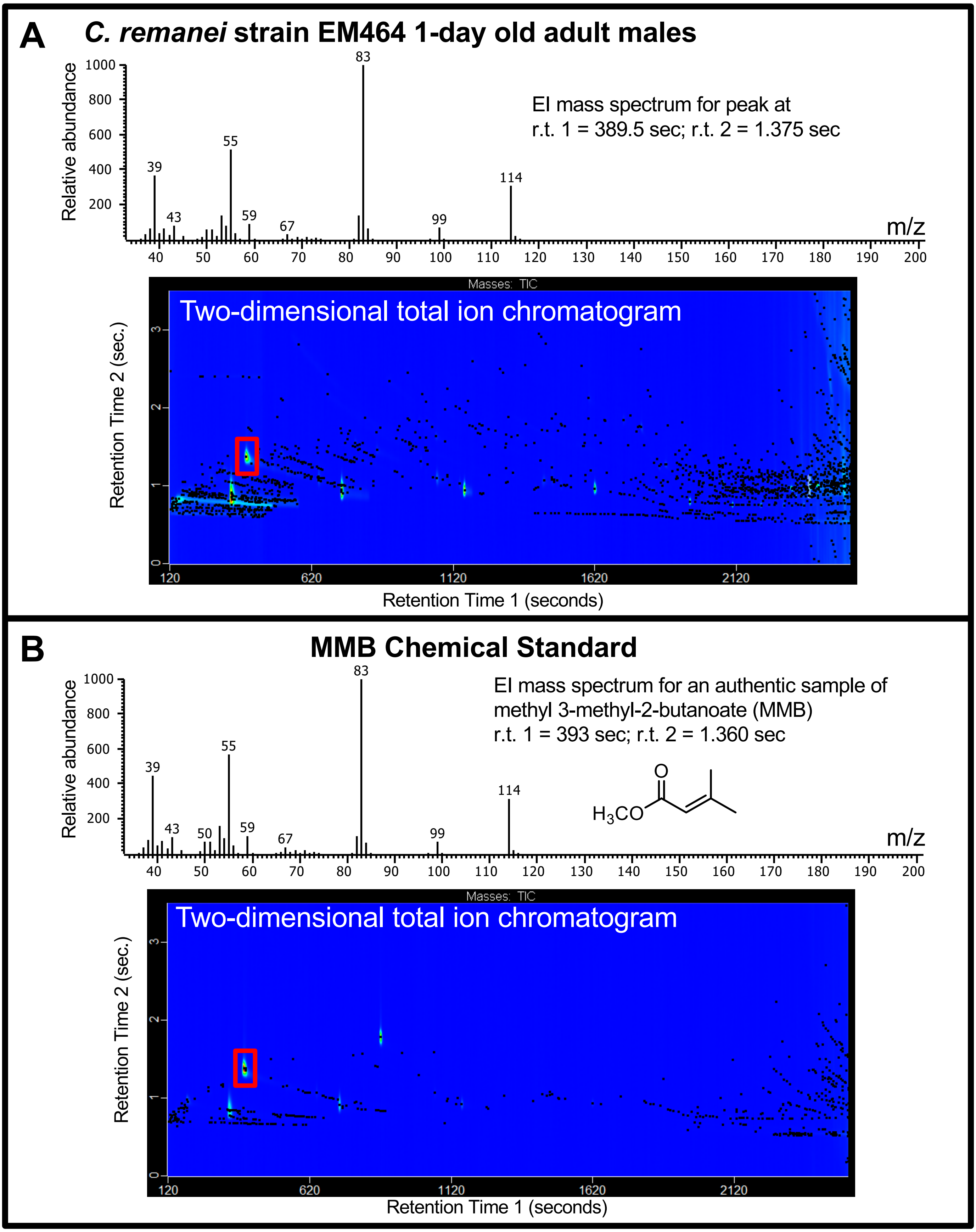

Supplementary Figure 1: SPME headspace volatile sampling followed by GC x GC – TOFMS analyses revealed that MMB is produced by *C. remanei* adult males. **(A)** EI mass spectrum and chromatogram (contour view) showing a chromatographic peak (indicated by red rectangle) which was unique to *C. remanei* adult male samples. **(B)** Mass spectrum and chromatogram acquired for an authentic sample of MMB. Black dots represent discrete elution peaks identified during data processing (shown for a signal-to-noise threshold of 30).

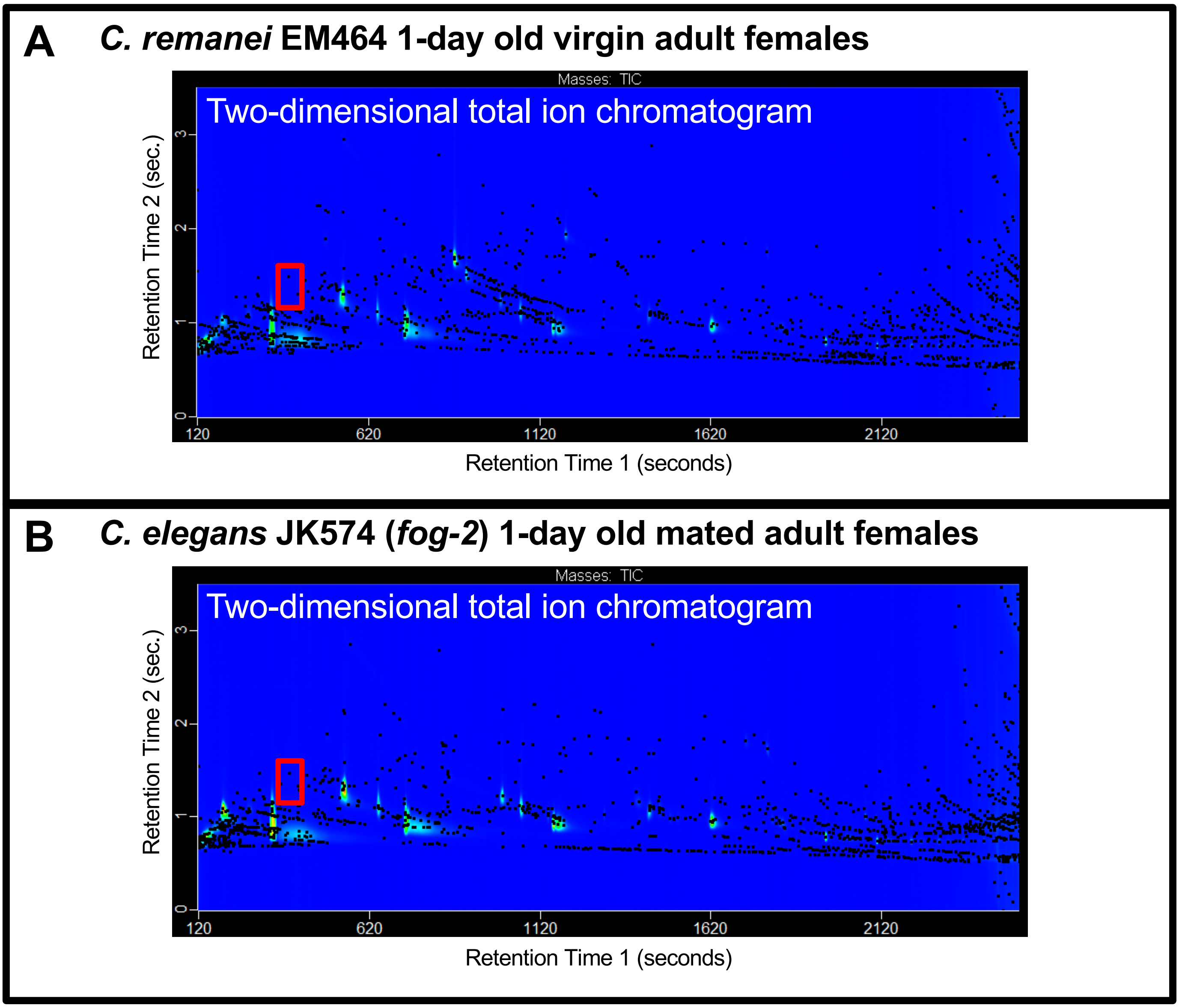

Supplementary Figure 2: MMB was not detected for *C. remanei* 1-day old virgin adult females, nor for *C. elegans* 1-day old mated adult females. GC x GC - TOFMS chromatograms acquired for SPME headspace volatile samples collected from *C. remanei* 1-day old virgin adult females **(A)**, and *C. elegans* 1-day old mated adult females **(B)**. The red rectangles indicate the retention time region within which MMB would elute, if present.

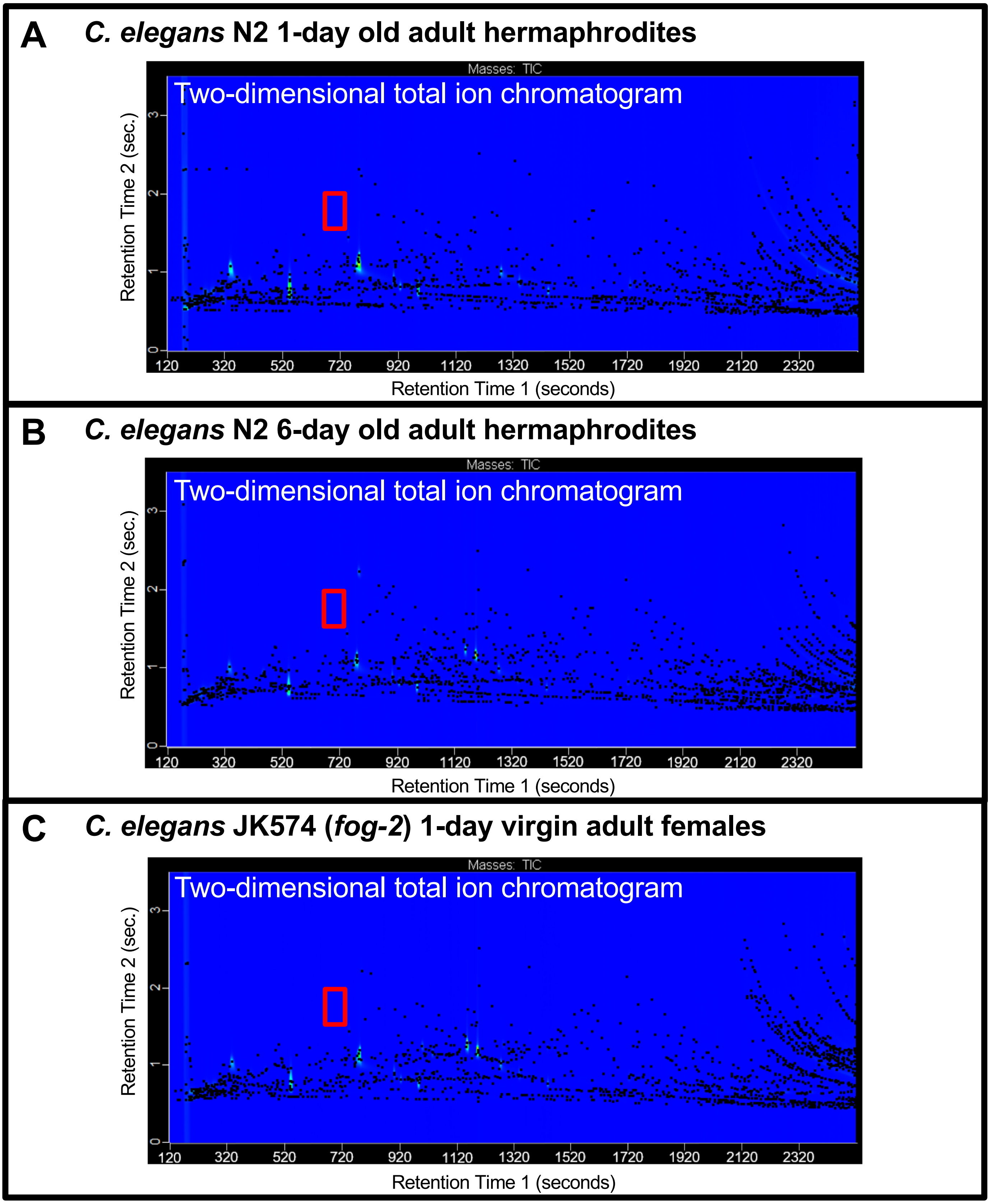

Supplementary Figure 3: MMB was not detected for any *C. elegans* sample. GC x GC - TOFMS chromatograms acquired for SPME headspace volatile samples collected from *C. elegans* N2 1-day old adult hermaphrodites **(A)**, *C. elegans* N2 6-day old adult hermaphrodites **(B)**, and *C. elegans* JK574 (*fog-2*) 1-day virgin adult females **(C)**. The chromatograms shown in this figure were acquired using an alternate secondary column (SGE Analytical Science BPX 50; described in Methods section), resulting in a change in the retention time region within which MMB would elute, if present (indicated by red rectangle).

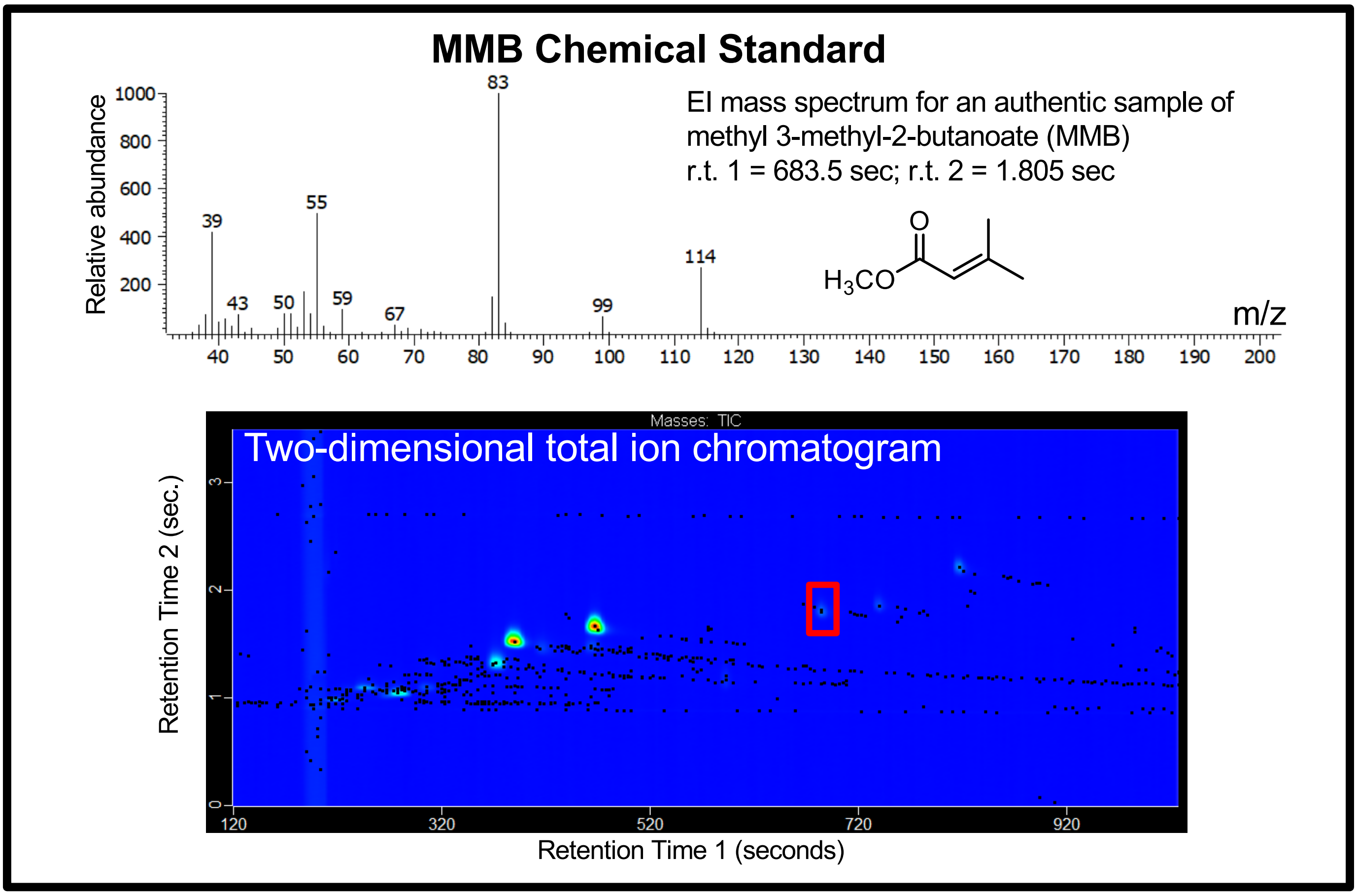

Supplementary Figure 4: Mass spectrum and chromatogram acquired for an authentic sample of MMB acquired using the alternate secondary column shown for Figure S3. For this analysis mass-spectral detection was stopped following the elution of the MMB standard (shown at the center of the red rectangle), resulting in a truncated range for Retention Time 1 compared to that shown in Figure S3.

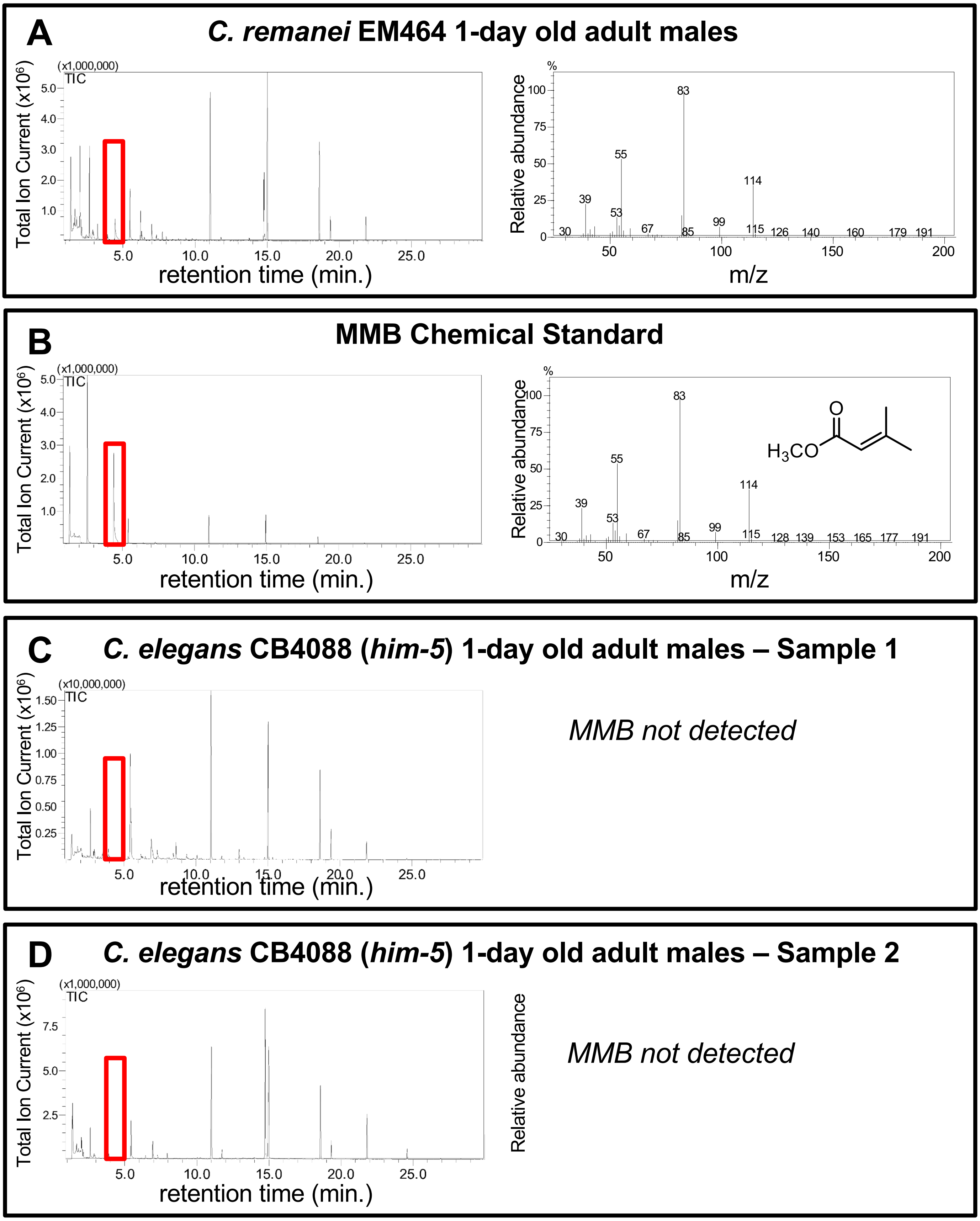

Supplementary Figure 5: Additional analyses by SPME-GC-MS confirmed the presence of MMB for *C. remanei* adult male samples, as well as the absence of MMB for *C. elegans* adult males. The red rectangle shows the retention time region within which MMB elutes, if present.

Supplementary Figures

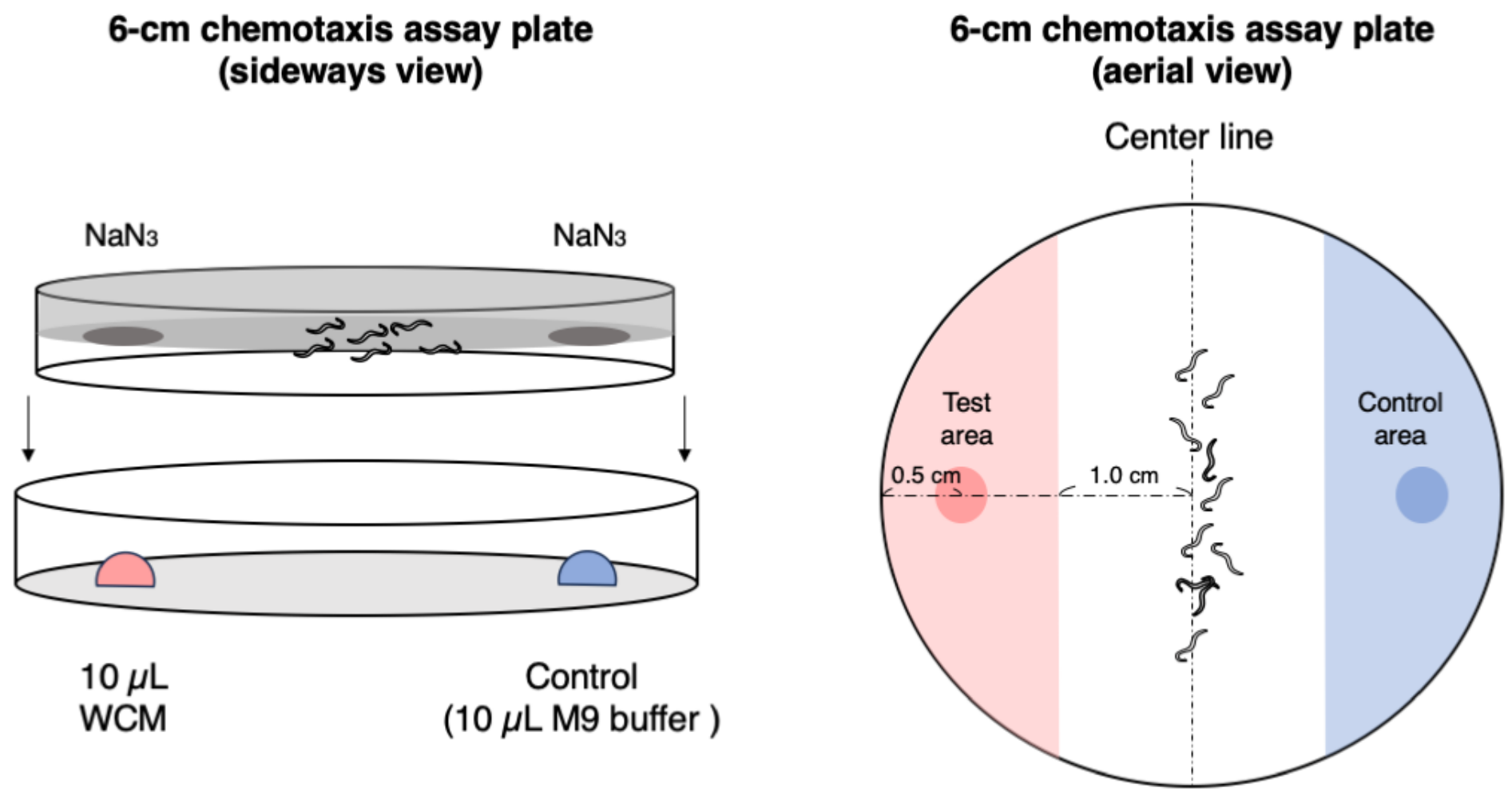

Supplementary Figure 6: Schematic diagram of chemotaxis assay with worm conditioned media (WCM) on the 6-cm chemotaxis assay plate.

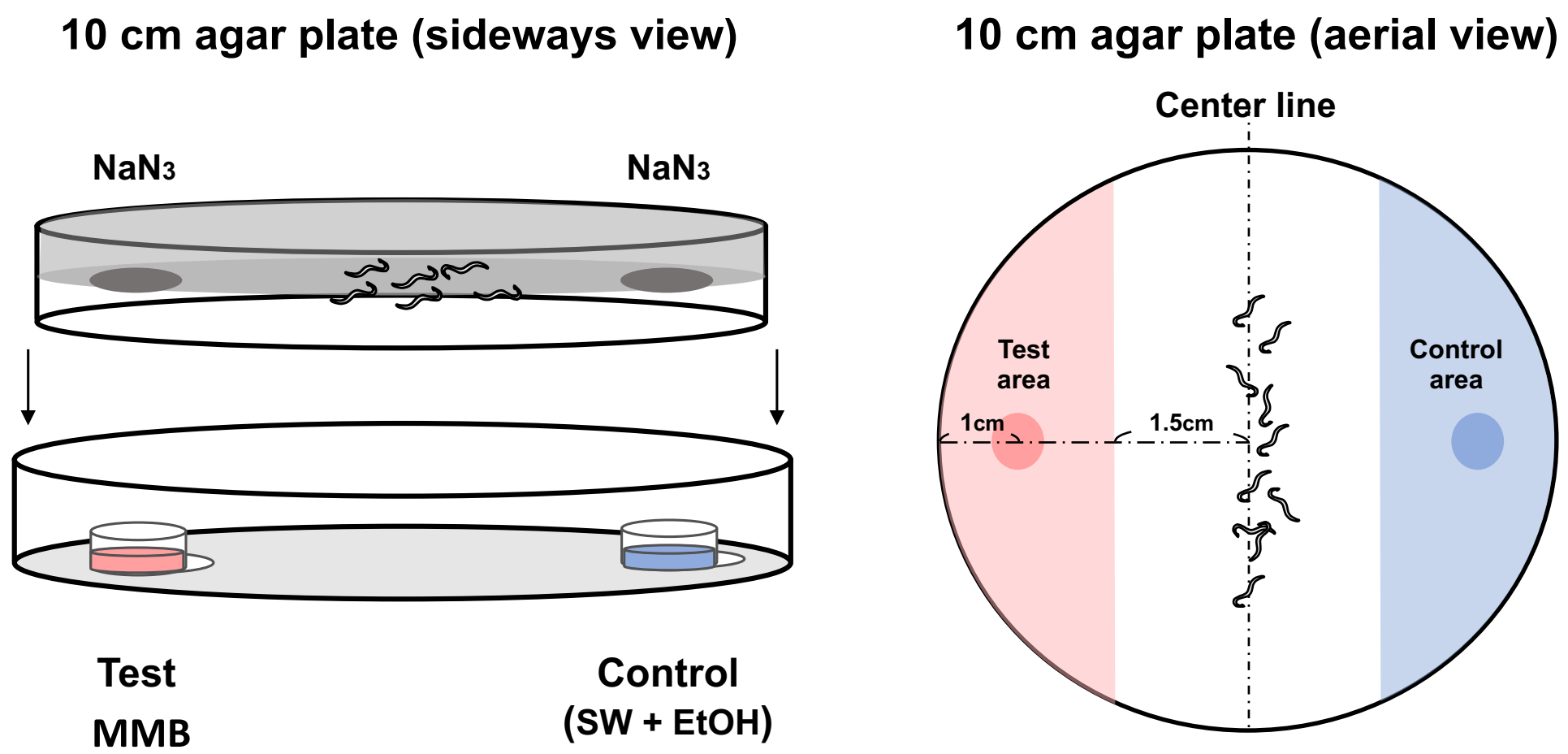

Supplementary Figure 7: Schematic diagram of chemotaxis assay with MMB on the 10-cm chemotaxis assay plate.
