# Supplementary material for: A male-derived volatile sex pheromone in Caenorhabditis nematodes identified through its mimicry by a predator
Source: Commun Biol. 2026 Jul 1;9:1382. doi: 10.1038/s42003-026-10575-4 (PMC13687015; doi:10.1038/s42003-026-10575-4)
Supplement: Supplementary file 3 [file 42003_2026_10575_MOESM2_ESM.docx]

**Description of Additional Supplementary Files**

**File name:** Supplementary Data 1

**Description:** Source data for the chemotaxis assays shown in Fig. 2A.

**File name:** Supplementary Data 2

**Description:** Source data for the chemotaxis assays shown in Fig. 2B.
